# The Abiotic Chemistry of Thiolated Acetate Derivatives and the Origin of Life

**DOI:** 10.1038/srep29883

**Published:** 2016-07-21

**Authors:** Kuhan Chandru, Alexis Gilbert, Christopher Butch, Masashi Aono, H. James Cleaves

**Affiliations:** 1 Earth-Life Science Institute, Tokyo Institute of Technology, 2-12-IE-1 Ookayama, Meguro-ku, Tokyo 152-8551, Japan; 2 Institute for Advanced Study, Princeton, NJ 08540, USA; 3 Blue Marble Space Institute of Science, 1515 Gallatin St. NW, Washington, DC 20011, USA; 4 Center for Chemical Evolution, Georgia Institute of Technology, Atlanta, GA 30332 USA

## Abstract

Thioesters and thioacetic acid (TAA) have been invoked as key reagents for the origin of life as activated forms of acetate analogous to acetyl-CoA. These species could have served as high-energy group-transfer reagents and allowed carbon insertions to form higher molecular weight compounds such as pyruvate. The apparent antiquity of the Wood-Ljungdahl CO_2_ fixation pathway and its presence in organisms which inhabit hydrothermal (HT) environments has also led to suggestions that there may be a connection between the abiotic chemistry of compounds similar to TAA and the origins of metabolism. These compounds’ apparent chemical simplicity has made their prebiotic availability assumed, however, although the kinetic behavior and thermochemical properties of TAA and analogous esters have been preliminarily explored in other contexts, the geochemical relevance of these compounds merits further evaluation. Therefore, the chemical behavior of the simplest thiolated acetic acid derivatives, TAA and methylthioacetate (MTA) were explored here. Using laboratory measurements, literature data, and thermochemical models, we examine the plausibility of the accumulation of these compounds in various geological settings. Due to the high free energy change of their hydrolysis and corresponding low equilibrium constants, it is unlikely that these species could have accumulated abiotically to any significant extant.

Several models for the origin of life invoke reactions occurring in the vicinity of volcanic or HT vent systems^1^,^2^,^3^. These are favored sites for the origin of life as they can be locations where considerable chemical disequilibrium is present, and high activation energy barriers to reaction, which are presently carried out by highly-evolved enzymes, may be overcome by the high temperatures present in such environments. High-energy compounds such as CO, COS, thioacids (R-COSH) and thioesters (R-COS-R′) are central to many of these schemes as feedstocks for primitive self-complexifying chemical cycles and the polymerization of biomolecules^4^,^5^,^6^,^7^,^8^,^9^,^10^,^11^,^12^. These high-energy compounds are generally suggested to be derived from reactions with equilibria that are more favorable at higher temperatures^13^, though synthesis of some of the components of coenzyme A has been achieved at lower temperatures^14^. While the chemistry of CO and CO_2_ has received detailed attention in the context of equilibrium HT vent chemistry^15^,^16^, even the simplest thioacids and thioesters, such as thioacetic acid (TAA) and methyl thioacetic acid (MTA), have received considerably less. A notable exception is the work of Bracher *et al.*^17^ who compared the competition of transthioesterification and hydrolysis for thioesters. These authors concluded that near pH 7 and 25 °C, given sufficiently high initial concentrations of reactants, exchange between thioesters can compete with hydrolysis. This analysis, however, ignores questions of the *sources* of these compounds, and important aspects of the abiotic geochemistry of TAA, MTA and their homologs warrant detailed consideration.

Several origin of life scenarios hypothesize that TAA, MTA or other thioacetate derivatives could have acted as acetyl-CoA analogues, donating acetate groups for organosynthesis in a primitive reverse tricarboxylic acid-(rTCA)-like cycle^2^,^6^,^18^,^19^ or as activating agents for carboxyl groups for transacylation reactions^7^,^10^,^20^. Conversion of geologic H_2_ and CO_2_ to CO through the water-gas shift reaction^21^, is often assumed to supply essential source materials for nascent biology. CO can be reduced to CH_4_ under geochemically plausible conditions, presumably via other intermediate oxidation-state C1 compounds such as HCHO and CH_3_OH^15^, the latter of which is also abundant in some geological settings, particularly in “serpentinizing systems,” in which reduced iron is oxidized by water yielding higher oxidation state iron oxides and H_2_ (Fig. 1)^22^.

CO has a high affinity for transition metals^23^, and surface catalysis is proposed to play a major role in several origin of life schemes involving thioesters, though sometimes the particular mineral is also a reagent in that it supplies reducing equivalents (*e.g.*, chemistries involving the FeS-FeS_2_ redox couple^24^). Surface bound organics are then proposed to undergo carbonyl-insertion reactions, which have been experimentally verified to occur under laboratory conditions^25^, in a process which could ultimately give rise to autocatalytic chemical cycles which depend on thioesters^2^.

However, as TAA and thioesters are “high-energy” compounds, in that their hydrolysis is very thermodynamically favorable; their formation is then *un*favorable, though the problems that may preclude their formation may be kinetic rather than thermodynamic, as we show here.

Interest in thioester chemistry due to the importance of CoA derivatives in biochemistry led to several controlled studies of their physical chemistry^26^,^27^,^28^. For example, TAA hydrolysis to acetic acid over a wide range of pH and temperature values has been explored to allow comparison with CoA hydrolysis. ΔG and E_a_ values for this reaction are available in the chemical literature^26^,^28^.

We examine here the equilibrium and kinetics of formation and hydrolysis of TAA and MTA using literature data corroborated and bolstered with experimental data over a wide geochemically relevant set of conditions. The abiotic chemistry of thioesters and their possible occurrence in natural HT environments is then discussed in the context of prebiotic chemistry.

## Materials and Methods

Acetic acid (99.99%), sodium methanethiolate (95%), Na_2_S**·**9 H_2_O (98%), NaHS**·**x H_2_O, MTA and TAA (96%, with the principal congener being acetic acid as determined by ^1^H NMR spectroscopy) were purchased from Sigma-Aldrich and used without further purification. D_2_O (99.9%) was purchased from Wako. Water (18.2 MΩ at 25 °C) was obtained from a Milli-Q Integral 3 Water Purification system.

In a typical experiment, TAA or MTA were mixed with 4:1 H_2_O/D_2_O to give a final concentration of 0.36 M or 0.036 M before the solution was placed into an NMR tube under air. A 0.1 M sodium carbonate buffer was used for pH 10 reactions and a 0.1 M sodium phosphate buffer was used for pH 7 reactions. 0.1 M phosphoric acid was used to adjust the pH of reactions conducted at pH 2.5. The natural pH of a 0.36 M aqueous TAA solution was measured as 2.5, increasing to ~3.8 after complete hydrolysis.

NMR analysis was begun within 10 minutes of preparing the mixtures. ^1^H NMR experiments were conducted in 5 mm outer diameter borosilicate and constricted NMR tubes (Norell Standard series) using a Bruker Avance III spectrometer. Temperature was held constant during measurements within +/−0.1 °C using a variable temperature unit. The pH of the reactions was monitored by placing aliquots of the reaction mixtures on Sigma-Aldrich Hydrion Brilliant disposable pH sticks, which are accurate to +/−0.1 pH unit. pH was further measured using a Mettler Toledo HM-25R pH meter. Reported pH values were not adjusted for the presence of D_2_O, which may introduce an inaccuracy of up to ~0.4 pH units at 25 °C^29^. Ionic strength was not explicitly controlled, but this has been found to have a relatively minor effect on TAA hydrolysis rates^28^.

Density Functional Theory (DFT) calculations were performed using the Jaguar 8.9 software package purchased from Schroedinger, Inc.^30^ with the B3LYP/6–31G* basis set. The modified Scaled Quantum Mechanical (SQM) Force Fields method^31^ was used to scale vibrational frequencies to improve calculations of thermochemical properties which were computed over 273.15 K to 573.15 K. Any remaining errors in absolute Gibbs free energies should be symmetrical and thus be cancelled out in calculation of the reaction ΔG values shown in Table 1. Energy of solvation in water was calculated using the Poisson Boltzmann Solvation model^32^ with energies of solvation included in the reported free energies. Frequencies less than 10 cm^−1^ were omitted from energy calculations for consistency with the harmonic approximation used for calculation of thermochemical properties^30^.

## Results

### Kinetics of TAA and MTA Hydrolysis

TAA or MTA hydrolysis leads to the formation of acetic acid (CH_3_COOH or AcOH) and H_2_S or CH_3_SH (Equation 1):


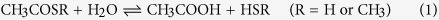


**Figure 2:**
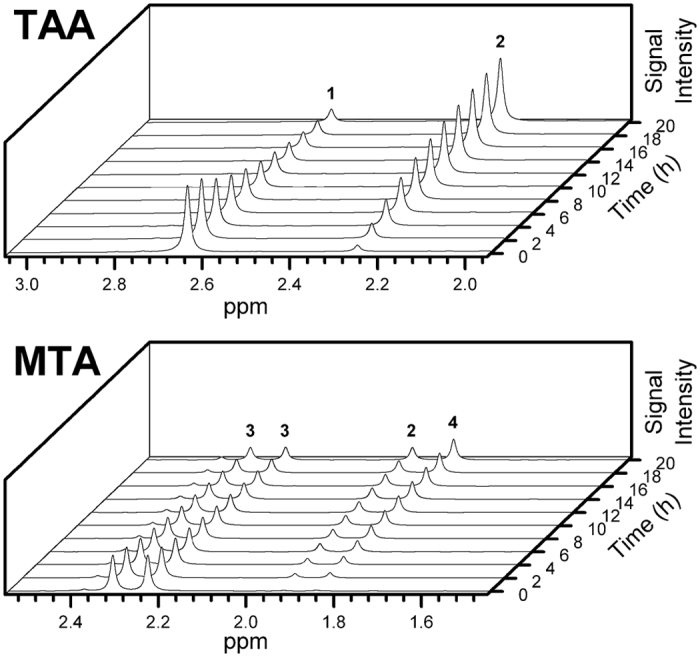
^1^H NMR spectra showing the time evolution of hydrolysis of TAA at 50 °C and pH 2.5 (top) and MTA at 30 °C and pH 10 (bottom). Peaks were identified by comparison with commercial standards: 1 TAA, 2 acetic acid, 3 MTA, 4 CH_3_SH. Integrated peak areas were used to determine kinetic parameters.

TAA and MTA hydrolysis and equilibrium was examined directly in real time by ^1^H NMR (Fig. 2).

**Figure 3:**
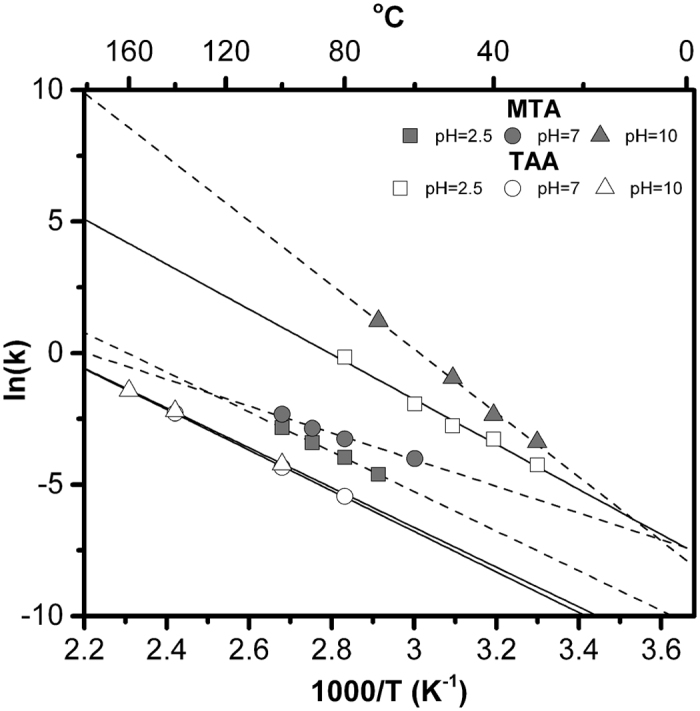
Arrhenius plots for the measured hydrolysis rates of TAA and MTA as a function of pH based on a pseudo-first order kinetic model.

Acetic acid was the only observable product of TAA degradation by ^1^H NMR. Oxidative coupling to generate disulfides was not observed in any experiment. TAA and MTA hydrolysis was fit to a pseudo-first order kinetic model, which gave rates consistent with those previously measured to within ±9.8%^28^. Measured TAA and MTA hydrolysis rates and corresponding half-lives as a function of temperature at pH 2.5, 7 and 10 are shown in Fig. 3. Arrhenius plots for these reactions measured between 30 and 160 °C gave activation energies of 71.2 ± 14.6 kJ mol^−1^ (pH 2.5), 64.2 ± 19.9 kJ mol^−1^ (pH 7) and 62.9 ± 22.7 kJ mol^−1^ (pH 10) for TAA. For comparison, Cefola *et al.*^26^ determined a value of 82.8 kJ mol^−1^ between 70 and 90 °C at pH 12.5. Our measured values for MTA between 30 and 100 °C at pH 2.5, 7 and 10 are 62.8 ± 3.2 kJ mol^−1^, 42.3 ± 14.9 kJ mol^−1^ and 101.2 ± 16.4 kJ mol^−1^, respectively.

The speciation of acetic acid, H_2_S and CH_3_SH are pH-dependent. At 25 °C the pK_a_ of acetic acid is 4.75, those of H_2_S are ~7 and >19 ± 2, and that of CH_3_SH ~10.4^33^,^34^. Hipkin and Satchell^28^,^35^ measured TAA hydrolysis between 40 and 90 °C as a function of pH and ionic strength, and found that acid-catalyzed TAA hydrolysis is expectedly second order at highly acidic conditions.

TAA is relatively unstable under acidic conditions, while MTA is least stable in base, which is explicable by the fact that the pK_a_ of the sulfhydryl group of TAA is 3.7, rendering it less electrophilic above this pH value, and because S^2−^ is a poor leaving group. Under the alkaline (~pH 10), cooler (~40 °C) conditions often favored for origin of life models for off-axis serpentinizing environments, TAA has a half-life of a few years, while that of MTA is a few hours.

It is worth noting that the dielectric constant of water drops by ~20–30% between the lowest and highest temperature points explored here for each compound and set of pH values^36^, nevertheless the Arrhenius plots are linear over this range.

### Thermodynamics of TAA and MTA Hydrolysis

DFT calculations at the B3LYP/6-31G* level were used to obtain Gibbs energies of formation for aqueous TAA, MTA, and their hydrolysis products. These values were then used to calculate the ΔG of hydrolysis for each species between 0 and 300 °C. The change in protonation state of the reactants and products with changing pH was also considered. The results of these calculations are shown in Table 1.

**Figure 4:**
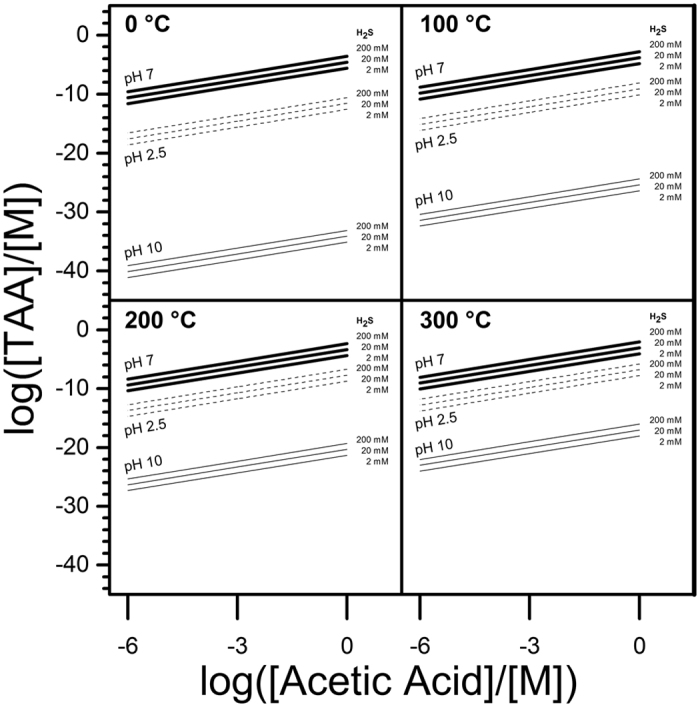
Calculated molar (M) equilibrium TAA concentrations at 0 to 300 °C from varying concentrations of acetic acid and ΣH2S. Equilibrium ΣH_2_S concentrations are given above the diagonal isobars.

For comparison, some measured free energies of hydrolysis of other simple thioesters are provided here: S-propyl-thioacetate, −32.2 kJ mol^−1^ 37, thiobenzoate, −32.2 kJ mol^−1 ^27, and acetyl CoA, −35.6 kJ mol^−1^ 38. The calculated ΔG of −51.7 kJ mol^−1^ for TAA at 0 °C and pH 2.5 gives an equilibrium constant for TAA synthesis from acetic acid and H_2_S of *~*1.3 × 10^−10^, strongly favoring hydrolysis. At higher temperatures the equilibrium constant is somewhat higher: at 300 °C it is ~7.3 × 10^−6^ (Fig. 4).

Given these low equilibrium constants, experimentally tracking the formation of TAA from acetic acid and H_2_S was experimentally difficult. Attempts were made to measure the formation of TAA and MTA from acetic acid and H_2_S or CH_3_SH at pH 2.5 between 30 to 60 °C, however no TAA or MTA was observable, even using very high initial concentrations of acetic acid and H_2_S or CH_3_SH. Our estimated ^1^H NMR detection limit for TAA was ~1 μM, corresponding to a K_eq_ of <5.5 × 10^−5^ using 1 M acetic acid and 1 M H_2_S.

As the non-detection of TAA could also have been due to sluggish kinetics, we attempted to estimate the time to equilibration based on the equilibrium constant and hydrolysis rate for pH 2.5. The rate of the forward reaction can be estimated using Equation 2:


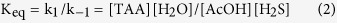


where k_1_ and k_−1_ are the rate constants for TAA formation and TAA hydrolysis, respectively. Using the measured pseudo-first order k_−1_ rate constant of 1.43 × 10^−2^ M^−1^ h^−1^ at 30 °C and pH 2.5, the forward rate constant (k_1_) at can be no larger than ~2.8 × 10^−10^ M^−1^ hr^−1^, and were it that high, it would not have been detectable during the time course of our experiments.

The linearity of the Arrhenius plots presented in Fig. 3 indicates a single mechanism under each investigated set of experimental conditions. The linearity of the Arrhenius plots is further strongly indicative of a single rate limiting step. To the extent this is true, the equilibrium approximation presented in Equation 2 is valid. Moreover, the equilibria as calculated are based on the DFT activation energies for each step, which are microscopically reversible, though they may not portray the entire speciation.

## Discussion

### Implications for TAA and MTA in Prebiotic Chemistry

Modern submarine HT systems are diverse, displaying extreme variability in the composition of their effluent depending on their host mineralogies and hydrodynamics, which have been studied extensively (Table 2 and references provided therein). Effluents range in temperature from several hundred degrees C to the temperature of ambient seawater (~2 °C), and in pH from ~2 to >10 12.

A variety of low molecular weight organic species have been detected in HT vent effluents^39^,^40^, including various hydrocarbons, as well as variable amounts of formic acid (Table 2). Formic acid may be derived from the hydration of CO^41^, which can itself can be derived from the water-gas shift reaction of CO_2_ and H_2_, both of which are abundant in some HT settings (Table 2)^41^.

At least some of the simple organics detected in modern vent effluents are generally agreed to have an abiotic origin based on their isotope systematics, though it is also acknowledged that this signal can be difficult to discern for other organics^42^,^43^. Still other organics have been convincingly determined to be of biological origin. For example, the isomer distribution of amino acids detected in several HT vent fluids from the Izu-Bonin Arc strongly suggests a biological origin^44^.

Despite laboratory experiments demonstrating the production of MTA from CO and CH_3_SH^25^, evaluation of modern HT vent effluents has not provided evidence for abiotically derived CH_3_SH^45^ or acetate^39^. CH_3_OH, the hydroxy analogue of CH_3_SH, does not appear to be a stable form of carbon under most HT vent conditions, being at best a transient intermediate in the inter-conversion between CO and CH_4_^15^. Thus the reaction of aqueous H_2_S and CH_3_OH is likely not a major contributor to environmentally measured CH_3_SH concentrations.

Sulfide concentrations in vent fluids are likely governed by concentrations of soluble metal species such as Ni and Fe, among others^46^. For example the K_sp_ values at 25 °C for FeS and NiS are 4.9 × 10^−18^ and 3 × 10^−21^ respectively^47^, with these values shifting as a function of temperature and pH. Though concentrations of sulfide (Σsulfide  =  H_2_S + SH^−^ + S^2−^) can be significant in high temperature fluids, these are precipitated rapidly upon cooling due to the formation of metal sulfides^48^. Thus Σsulfide speciation in modern HT systems has been measured variably between ~0 and a maximum near 20 mM (Table 2). In off-axis serpentinizing vent systems such as Lost City, sulfide concentrations are typically much lower, often less than 1 mM (Table 2) and the majority of S is often found in the form of sulfate, despite the fact that these vents often discharge considerable amounts of dissolved H_2_^45^.

With regard to the propensity of HT vent chemistry to produce organics beyond the simple hydrocarbons which may be produced in trace amounts in such systems^43^, it is worth noting that HT systems chemistry may be self-regulating in many respects. High H_2_ systems, which may contain large amounts of CH_4_, may inherently limit the production of higher hydrocarbons due to equilibria such as those obtained in Equation 3^49^.


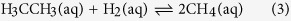


The estimated ΔrG′° for this reaction is −68.9 ± 14.2 kJ mol^−1^ 50, giving K_eq_ values of ~1.2 × 10^12^, 4.4 × 10^9^ and 4.0 × 10^7^ at 25, 100 and 200 °C, respectively.

Likewise, high CO_2_/reduced Fe systems are limited in their ability to supply H_2_ or reduced C (including CO, which appears to be the active species for Fischer Tropsch Type synthesis)^16^ due to the precipitation of siderite (FeCO_3_)^51^. Thus, fluids with high dissolved inorganic carbon limit the ability of Fe^2+^ to serve as a reducing agent, even though high concentrations of C species might allow for escape from the kinetic limitations of forming higher C-species.

If the upper mantle had already largely reached its modern oxidation state by 4.35 Ga, as appears to be true from recent measurements of rare earth elements in zircons^52^ then modern HT vent chemistry is likely similar to what one could expect for early Earth geochemistry^12^,^53^, though the composition of bulk seawater may have been markedly different. The heat flux on the primitive Earth was likely somewhat higher, making mean oceanic circulation times through seafloor HT vent systems on the whole shorter^54^, nevertheless the residence times of fluids at high temperatures would still be on average long enough to ensure equilibration of seawater with host mineralogies. There is little evidence that mantle-derived rocks have ever contained markedly higher C or S concentrations^55^,^56^, though there have been significant changes in bulk ocean water composition over time^57^. pH and Fe, C, and S species concentrations in the oceans between 3.5 Ga and 4.4 Ga are poorly constrained due the lack of corroborating geological evidence.

Nevertheless, high- and low-end estimates for the concentrations of some species can be broadly constrained. Sulfide concentrations in the bulk Archaen ocean (~4-2.5 Ga) have been suggested to have been in the low mM range^48^, which is similar to concentrations observed in modern HT vent fluids (Table 2).

For the sake of subsequent calculations, we will assume a generous 20 mM sulfide concentration for vent fluid or the bulk ocean, acknowledging that this value may be overestimated by orders of magnitude depending on the exact geochemical setting under consideration. Due to metal sulfide solubility product constraints, these values are unlikely to be *underestimated* in any environment.

Equilibrium TAA concentrations would be limited by H_2_S concentrations, MTA concentrations similarly by CH_3_SH concentrations. Except under strongly acidic conditions, TAA is generally more stable than MTA, but TAA is undoubtedly more plausible simply due to the likely much higher abundance of H_2_S, which is more abundant than CH_3_SH in modern HT vent fluids by a factor of 1000 or more (Table 2). It is difficult to envision a geochemical setting in which H_2_S concentrations are lower than CH_3_SH concentrations, due to the equilibria involved in making CH_3_SH in the first place^58^.

Figure 4 shows estimates for maximal equilibrium concentrations of TAA in various HT settings, as a function of possible ΣH_2_S and Σacetate concentrations, and using the K_eq_ values for TAA formation derived from the calculated free energy values in Table 1. Much of the area shown in this figure represents parameter combinations which are unlikely to be attainable in natural HT systems. Measured concentrations (Table 2) of acetate and H_2_S in fluids from Lost City^39^ and hydrothermally altered biologically organic-enriched sediments from Guaymas basin^59^ would yield TAA concentrations of 1.4 × 10^−49^ and 2.1 × 10^−11^ M, respectively. These rough equilibrium calculations do not take into account potential competing side reactions (which could only lower these values) and mineral or other types of catalysis (which in principle would only speed the time to equilibration), nevertheless the equilibria cannot be high due to the factors discussed above.

Given the second order rate and yield dependencies of condensation reactions, which typically decline inversely as a function of reactant concentrations, these concentrations are very low for the sake of performing more complex organic chemistry in HT environments. In our study, even examining the behavior of TAA or MTA concentrated far beyond what is plausible in natural marine environments, we note that only the predicted hydrolysis products are observed by NMR.

Huber and Wächtershäuser^25^ reported ~0.5% acetate yields based on input CH_3_SH (8 mM) in the presence of 350 mM CO. This is ~500 times and ~3700 times the highest CH_3_SH and CO concentrations respectively measured to date in a natural vent fluid^45^. All isotopic measurements to date suggest that acetate detected in HT vent fluids is derived from biological material^39^ despite being predicted to form abiotically under HT conditions^13^.

Reduced carbon species in modern HT vent fluids have not been measured outside of the mM range (Table 2), and it is clear that in some cases, for example the Guaymas Basin, high organic contents are due to the liberation of biological material from sediments^59^. These fluids contain the highest concentrations of acetate measured in submarine HT fluids to date, but acetate is a common component of naturally cracked petroleum^60^.

The redox equilibrium between H_2_ released by serpentinization reactions and one-C species can be constrained. C-C bond forming reactions are most kinetically favorable from formate/CO and as would be expected the equilibrium between CO and formate, a simple hydration, is obtained rapidly, while that between CO_2_ and CH_4_ is not^15^. Thus CO (or its hydrate) is likely the limiting reagent for higher hydrocarbon formation.

Previous models of HT origins of life have appealed to MTA as opposed to TAA based on experiments by Heinen and Lauwers^61^ and Huber and Wächtershäuser^25^ in which CH_3_SH and MTA were prepared from very high concentrations of reagents. Huber and Wächtershäuser’s^25^ demonstrated synthesis of acetate from CH_3_SH, CO, and NiSO_4_ or various metal sulfides was proposed to proceed through MTA as an intermediate. However, the conditions of the experiment are of questionable geochemical relevance: for example, 160 mM Na_2_S was employed, which is much higher than that observed in any extant natural system (Table 2).

Concentration processes would be necessary for the prebiotic synthesis of these compounds. Potential concentration mechanisms such as thermophoresis^62^ deserve much further experimental investigation in this context. This has become a major touchstone of models for the origin of life in HT vent systems^12^,^63^, though to date little evidence has been presented which suggests that small molecules such as TAA can be concentrated by several orders of magnitude by this mechanism in geochemical settings.

Dissolved Fe^2+^ catalyzes TAA hydrolysis, while solid FeS had no effect under the same conditions^64^. Nevertheless, mineral surface catalysis and catalysis by dissolved organic and/or inorganic species are unlikely to solve these instability problems. TAA and MTA hydrolyze rapidly, and their formation equilibria are very low, thus little is gained by achieving equilibrium more rapidly through catalysis, and for group transfer from very low concentration transient species the resulting acylates would have to be extremely stable to accumulate.

The effect of pH on the equilibrium constants for TAA derivatives may be attributable to pK_a_ differences between the reacting acid and thiol species, as has been suggested in the case of amide bond formation^65^. Simply based on the pK_a_ differences between H_2_S (~7.0) and CH_3_SH (~10.4) with acetic acid (~4.8) at 25 °C, one would expect the K_eq_ for TAA to be significantly higher than for MTA, which is concordant with our DFT calculations. For reference the pK_a_’s of ethane thiol (~10.6) and butane thiol (~10.5) are fairly close to those reported for CoASH (~9.6–10)^33^, thus there is no strong reason to think that higher thiols would have significantly different K_eq_ values, and the abundances of higher thiols can reasonably be expected to be much lower.

While the pH of vent fluids has probably always been locally controlled, the pH of the prebiotic oceans remains uncertain. The best geochemists have been able to infer is a minimum pH for the oceans, which is on the order of 5–6^66^, though some have argued for a pH closer to neutral^67^. A full explanation for the pH early oceans must account for buffering systems in addition to carbonate, for example marine clays^68^, and there is a general need to balance species such as Ca^2+^ and Mg^2+^. It is not clear that a high, neutral or low pH environment bulk ocean is necessarily preferable for the origin of life, though in scenarios which invoke TAA or MTA, each has significant consequences.

Acidic oceans do not seem to be preponderant on icy worlds in our solar system where measurement has been possible, for example on Enceladus, as inferred from mass spectrometry of plume ejecta^69^. The presence of significant amounts of dissolved ammonia has been implicated as necessary to render many icy moons’ subsurface water reservoirs liquid^70^. The early phases of Mars’ evolution, by most accounts when Mars was most likely to have hosted surface oceans, seem to be consistent with slightly basic to circum-neutral pH, though it appears that subsequent planetary evolution drove Mars’ surface into a more acidic regime^71^.

It is beyond the scope of this paper to examine every conceivable reaction mechanism which could lead to thioesters in hydrothermal environments. There are other routes by which TAA derivatives could be reasonably abiotically synthesized. For example the sulfhydrolysis of acetonitrile (ACN) with H_2_S to give TAA^10^. ACN is a product of abiotic atmospheric synthesis from reducing gas mixtures^72^. The primary intermediate of the reaction of H_2_S with ACN, thioacetamide, is also unlikely to accumulate in most environments, degrading principally to TAA under basic conditions, and acetamide under acidic ones^73^.

The addition of thiols to aldehydes followed by oxidation could also yield thioesters. However, this ignores the problem of thiol concentration already discussed and would require the presence of significant concentrations of aldehydes which are unlikely to be stable to the temperature and redox conditions prevailing in HT environments (see for example Nagai *et al.*^74^ and Schulte and Shock^75^; to our knowledge, there have been no reports to date of significant amounts of abiotic aldehydes in natural submarine HT environments). In any event the products would still suffer from the extreme hydrolytic instability demonstrated here. Such a mechanism may be more favorable in a low-temperature evaporative environment, but seems difficult in the context of a flow-through, elevated temperature marine environment.

## Conclusions

The origin of life almost certainly occurred during a period for which little is left of the primary geological record. Unfortunately this means a great deal is inferred in many scenarios. Fortunately, a good deal can be extrapolated based on what is measurable in the laboratory. Modern organisms are obviously capable of internally generating high-energy compounds such as thioesters under relatively extreme conditions by coupling their synthesis to other cellular energy-dissipating reactions, and shunting them for use in other biochemical pathways before they have time to decompose. The goal of this paper is to examine the abiotic generation and behavior of thioester derivatives in natural settings and by doing so, identify constraints for prebiotic chemistry and origin of life models. Until thioesters were capable of being generated at reasonable steady states by relatively complex chemical assemblages (e.g. systems on the continuum from chemical networks to fully recognizable biological systems), it is unlikely such compounds could have contributed to the origin or maintenance of such assemblages, except possibly in certain specialized environments as discussed below. TAA and MTA differ significantly in terms of their stability and reactivity as a function of pH and temperature, though both are implausible prebiotic reagents for different reasons in different contexts.

The variety of schemes in which TAA and MTA are implicated prompted this investigation. The large free energy changes for the hydrolysis of TAA and MTA have contributed to the notion of their potential utility as acetyl-CoA analogues in prebiotic chemistry^6^,^7^,^25^,^63^. Thioesters were implicated in the origin of life after the discovery of the importance of CoA in biochemistry and non-ribosomal peptide synthesis^7^,^76^. Early models were not generally tied to any particular geochemical setting, it was merely pointed out that these compounds were sufficiently energetic, and possibly stable enough, to allow for spontaneous group transfer, and that analogue compounds play important roles in contemporary biochemistry. More recent models suggest that TAA and thioesters are vestigial links between geochemical processes and the origin of intermediary metabolism, for example as reagents for a primitive Wood-Ljungdahl-like pathway^2^,^6^,^18^, based on the chemistry which may occur in natural settings and the conjecture that inorganic ligands used in contemporary biological systems, such as FeS clusters, are derived cognates of naturally occurring minerals^2^,^6^,^77^, *i.e.*, that life originally depended on specific environmental chemical pathways, which it gradually coopted.

In examining the kinetics of thioester exchange reactions, Whitesides and coworkers concluded that there was no *a priori* reason on the basis of the kinetics of hydrolysis for blanket skepticism or rejection of theories that invoke thioesters as molecules of prebiotic importance^17^. However, the kinetic and thermodynamic considerations presented here highlight difficulties which may exclude thioesters from consideration in many prebiotic contexts.

In considering the plausibility of the existence of any given species, and its possible concentrations, it is useful to adopt a systems-based analysis. The sources and sinks of species are crucial considerations, as are the temporal dynamics of material transport, and chemical kinetics and equilibria in geological settings.

It is difficult to categorically state that some species could have been present in any given concentration in any given environment on the primitive Earth, especially since there may have existed gradients of environmental parameters where many combinations of pH, temperature, and speciation could have been juxtaposed for variable amounts of time^78^. Nevertheless, one can ask questions about what combination of factors would have allowed for a set of conditions to have been common or even transiently achievable.

The instabilities and difficulty of synthesis of various organic compounds have been used previously to argue against the likelihood of various compounds’ accumulation and involvement in the origin of life in certain settings^79^. TAA derivatives are considerably less stable than most of the compounds examined in these papers and must be considered critically in that light, though they are not the most unstable compounds considered in HT settings. For example, acetylphosphate has a half-life of only ~1.2 hours between pH 2.7 and 7.7 even at 39 °C^80^.

A number of extant organisms carry out reactions which initially fix CO via the Wood-Ljungdahl pathway, ultimately yielding acetyl-CoA. However, while these CoA analogs could conceivably be generated in very low steady-states abiotically, they may be unlikely feedstocks for the origin of life or primitive biology. In this study, we reacted extremely high concentrations of TAA or MTA, and found that there are no products aside from the hydrolytic ones, thus there may be relatively few reactions by which acetate equivalents could be donated by TAA or its derivatives, aside from acetyl group transfer (although TAA is also known to act as an S nucleophile to alkenes^81^), and then only in settings which allowed for considerably higher concentrations of these compounds than HT vents are likely to provide. The flux of energy made available by these compounds would likewise be insignificant compared to that available from other compounds such as CO and H_2_.

Small equilibrium amounts of TAA derivatives obtained by quenching of high temperature fluids could be produced in HT vent settings and conducted to the bulk ocean, and then concentrated by evaporation to give higher concentrations of TAA, but such a mechanism requires the site of their use to be an evaporative environment rather than the vent itself.

Lastly, since these compounds are such important reagents in so many HT origin of life schemes, their remarkable instability under typical HT environment conditions suggests estimates of the ubiquity of suitable environments for the origin of life in and beyond our solar system may be somewhat overestimated^82^,^83^, if these compounds are indeed crucial for jump-starting early metabolism.

## Additional Information

**How to cite this article**: Chandru, K. *et al.* The Abiotic Chemistry of Thiolated Acetate Derivatives and the Origin of Life. *Sci. Rep.* **6**, 29883; doi: 10.1038/srep29883 (2016).

## Figures and Tables

**Figure 1:**
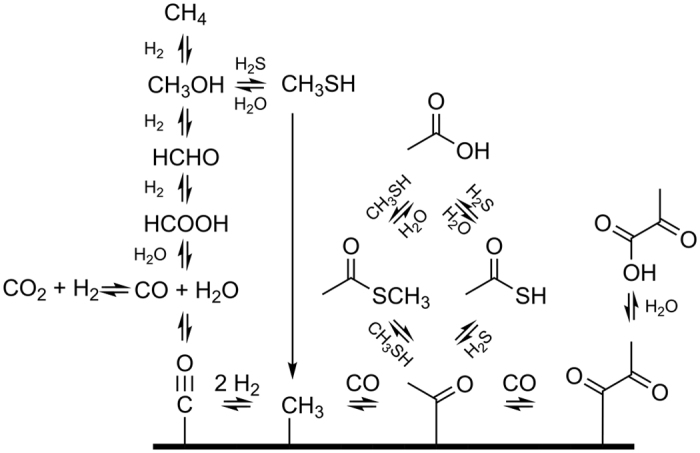
Some proposed mechanisms for the abiotic production of TAA and MTA in HT settings. The thick line represents a mineral surface (*e.g.*, a metal sulfide or oxide).

**Table 1:** Results of DFT calculations to estimate free energies of hydrolysis of TAA and MTA as a function of pH and temperature according to various reaction mechanisms.

pH	Reaction	ΔG_RXN_0 °C kJ mol^−1^	ΔG_RXN_100 °C kJ mol^−1^	ΔG_RXN_200 °C kJ mol^−1^	ΔG_RXN_300 °C kJ mol^−1^
2.5	TAA + H_2_O →AcOH + H_2_S	−51.7	−53.1	−54.4	−55.9
7	TAA^−^ + H_2_O → AcO^−^ + H_2_S	−15.2	−15.2	−15.1	−15.0
10	TAA^−^ + OH^−^ → AcO^−^ + SH^−^	−169.4	−169.2	−168.7	−168.2
2.5	MTA + H_2_O → AcOH + CH_3_SH	−35.3	−37.6	−39.8	−42.0
7	MTA + OH^−^ → CH_3_SH + AcO^−^	−163.7	−167.3	−170.6	−173.9
10	MTA + 2 OH^−^ → AcO^−^ + CH_3_S^−^ + H_2_O	−289.1	−293.3	−297.2	−300.8

**Table 2:** Measured physical parameters and concentrations of species relevant to this discussion in various marine HT vents and modern bulk seawater. ^a^Refs 43,45; ^b^Refs 84, 85, 86; ^c^Ref. 87, ^d^Refs 39,88, ^e^Ref. 59, ^f^Ref. 45, −.= not determined, bd = below detection limit as reported.

	Rainbow^a,c,d^	9°50′ East Pacific Rise^a,b,d^	Guaymas Basin^a,e^	Lost City^a,d,f^	Seawater ^a,b,c,d^
Temperature (°C)	365	221–363	172–299	94	2
pH	3	3.5–4.2	4.48–6.09	10.2	8.1
H_2_ (mM)	12–16	0.4–1.2	0.5–3.3	9.1–10.5	—
CO_2_ (mM)	20–24	8.19–99.3	35–51	<0.19	2.36
CO (μM)	5.1–7.3	<2	0–92	<1	—
CH_4_ (mM)	2	<0.11	44–59	<1.09	—
Formate (μM)	—	—	—	<140	bd
Acetate (μM)	—	—	65–365*	1–35	bd
H_2_S (mM)	2–3.3	4.6–19.2	6.8–8	0.07–0.47	—
CH_3_SH (nM)	7.7–10.3	4–11.4	8.1–16,700	1.4–1.9	—
SO_4_^2−^ (mM)	0	0	0	1–4	53

*The acetate detected in Guaymas Basin fluids is attributed to contributions from microbes and thermal cracking of biological material in sediments.

## References

[b1] Corliss J., Baross J. & Hoffman S. An hypothesis concerning the relationship between submarine hot springs and the origin of life on Earth. Oceanol. Acta 4 Supplement, 59–69 (1981).

[b2] Wächtershäuser G. Groundworks for an evolutionary biochemistry - the iron sulfur world. Prog. Biophys. Mol. Biol. 58, 85–201 (1992). 1509092 10.1016/0079-6107(92)90022-x

[b3] Russell M. J., Daniel R. M., Hall A. J. & Sherringham J. A. A hydrothermally precipitated catalytic iron sulphide membrane as a first step toward life. J. Mol. Evol. 39, 231–243, doi: 10.1007/bf00160147 (1994).

[b4] Cody G. Transition metal sulfides and the origins of metabolism. Ann. Rev. Earth Planet. Sci. 32, 569–599 (2004).

[b5] Wächtershäuser G. Evolution of the first metabolic cycles. Proc. Nat. Acad. Sci. USA 87, 200–204 (1990). 2296579 10.1073/pnas.87.1.200 PMC53229

[b6] Russell M. J. & Martin W. The rocky roots of the acetyl-CoA pathway. Trends in Biochemical Sciences 29, 358–363, doi: 10.1016/j.tibs.2004.05.007 (2004). 15236743

[b7] de Duve C. Clues from present-day biology: the thioester world. In The Molecular Origins of Life (ed. Brack A.) 219–236 (1998).

[b8] Huber C. & Wächtershäuser G. Peptides by activation of amino acids with CO on (Ni,Fe)S surfaces: Implications for the origin of life. Science 281, 670–672, doi: 10.1126/science.281.5377.670 (1998). 9685253

[b9] Hagan W. J. Uracil-catalyzed synthesis of acetyl phosphate: A photochemical driver for protometabolism. Chem Bio Chem. 11, 383–387, doi: 10.1002/cbic.200900433 (2010). 20039252

[b10] Liu R. & Orgel L. E. Oxidative acylation using thioacids. Nature 389, 52–54, doi: 10.1038/37944 (1997). 9288964

[b11] Nakajima T., Yabushita Y. & Tabushi I. Amino acid synthesis through biogenetic-type CO_2_ fixation. Nature 256, 60–61 (1975). 1134583 10.1038/256060a0

[b12] Martin W., Baross J., Kelley D. & Russell M. J. Hydrothermal vents and the origin of life. Nat. Rev. Micro. 6, 805–814 (2008). 10.1038/nrmicro1991 18820700

[b13] Shock E. L. & Schulte M. D. Organic synthesis during fluid mixing in hydrothermal systems. J. Geophys. Res. Planets 103, 28513–28527, doi: 10.1029/98JE02142 (1998).

[b14] Keefe A. D., Newton G. L. & Miller S. L. A possible prebiotic synthesis of pantetheine, a precursor to coenzyme A. Nature 373, 683–685, doi: 10.1038/373683a0 (1995). 7854449

[b15] Seewald J. S., Zolotov M. Y. & McCollom T. Experimental investigation of single carbon compounds under hydrothermal conditions. Geochim. Cosmochim. Acta 70, 446–460, doi: 10.1016/j.gca.2005.09.002 (2006).

[b16] McCollom T. M., Lollar B. S., Lacrampe-Couloume G. & Seewald J. S. The influence of carbon source on abiotic organic synthesis and carbon isotope fractionation under hydrothermal conditions. Geochim. Cosmochim. Acta 74, 2717–2740, doi: 10.1016/j.gca.2010.02.008 (2010).

[b17] Bracher P. J., Snyder P. W., Bohall B. R. & Whitesides G. M. The relative rates of thiol–thioester exchange and hydrolysis for alkyl and aryl thioalkanoates in water. Origins of Life and Evolution of Biospheres 41, 399–412 (2011). 10.1007/s11084-011-9243-4 21728078

[b18] Blöchl E., Keller M., Wächtershäuser G. & Stetter K. O. Reactions depending on iron sulfide and linking geochemistry with biochemistry. Proc. Nat. Acad. Sci. USA 89, 8117–8120 (1992). 11607321 10.1073/pnas.89.17.8117 PMC49867

[b19] Morowitz H. J. A theory of biochemical organization, metabolic pathways, and evolution. Complexity 4, 39–53, doi: 10.1002/(SICI)1099-0526(199907/08)4:6<39::AID-CPLX8>3.0.CO;2-2 (1999).

[b20] Weber A. L. & Orgel L. E. Formation of peptides From glycine thioesters. J. Mol. Evol. 13, 193–202 (1979). 501743 10.1007/BF01739479

[b21] Smith R., Loganathan M. & Shantha M. S. A review of the water gas shift reaction kinetics. Int. J. Chem. Reactor Eng. 8 (2010).

[b22] Jöns N. & Bach W. In Encyclopedia of Marine Geosciences (eds. Harff J., Meschede M., Petersen S. & Thiede J.) Ch. 119-1, 1–12 (Springer Netherlands, 2014).

[b23] Cody G. D. *et al.* Geochemical roots of autotrophic carbon fixation: hydrothermal experiments in the system citric acid, H_2_O-(±FeS)−(±NiS). Geochim. Cosmochim. Acta 65, 3557–3576, doi: 10.1016/s0016-7037(01)00674-3 (2001).

[b24] Wang W., Yang B., Qu Y., Liu X. & Su W. FeS/S/FeS_2_ redox system and its oxidoreductase-like chemistry in the iron-sulfur world. Astrobiology 11, 471–476, doi: 10.1089/ast.2011.0624 (2011). 21707387

[b25] Huber C. & Wächtershäuser G. Activated acetic acid by carbon fixation on (Fe,Ni)S under primordial conditions. Science 276, 245–247, doi: 10.1126/science.276.5310.245 (1997). 9092471

[b26] Cefola M., Peter S. S., Gentile P. S. & Celiano R. A. V. Rate of hydrolysis of thiolacetic acid in basic solutions. Talanta 9, 537–540, doi: 10.1016/0039-9140(62)80125-8 (1962).

[b27] Cox R. A. & Yates K. Mechanistic studies in strong acids. VIII. Hydrolysis mechanisms for some thiobenzoic acids and esters in aqueous sulfuric acid, determined using the excess acidity method. Can. J. Chem. 60, 3061–3070, doi: 10.1139/v82-438 (1982).

[b28] Hipkin J. & Satchell D. P. N. The spontaneous and acid-catalysed hydrolysis of thiolcarboxylic acids. Tetrahedron 21, 835–842, doi: 10.1016/0040-4020(65)80017-5 (1965).

[b29] Krżel A. & Bal W. A formula for correlating pK_a_ values determined in D_2_O and H_2_O. J. Inorg. Biochem. 98, 161–166 (2004). 14659645 10.1016/j.jinorgbio.2003.10.001

[b30] Bochevarov A. D. *et al.* Jaguar: A high-performance quantum chemistry software program with strengths in life and materials sciences. Int. J. Quant. Chem. 113, 2110–2142, doi: 10.1002/qua.24481 (2013).

[b31] Baker J., Jarzecki A. A. & Pulay P. Direct scaling of primitive valence force constants: an alternative approach to scaled quantum mechanical force fields. J. Phys. Chem. A 102, 1412–1424 (1998).

[b32] Marenich A. V., Olson R. M., Kelly C. P., Cramer C. J. & Truhlar D. G. Self-consistent reaction field model for aqueous and nonaqueous solutions based on accurate polarized partial charges. J. Chem. Theory Comp. 3, 2011–2033 (2007). 10.1021/ct7001418 26636198

[b33] Perrin D. D. Ionisation Constants of Inorganic Acids and Bases in Aqueous Solution. (Elsevier Science, 2013).

[b34] Hughes M. N., Centelles M. N. & Moore K. P. Making and working with hydrogen sulfide: The chemistry and generation of hydrogen sulfide *in vitro* and its measurement *in vivo*: a review. Free Radic. Biol. Med. 47, 1346–1353, doi: 10.1016/j.freeradbiomed.2009.09.018 (2009). 19770036

[b35] Hipkin J. & Satchell D. P. N. 189. Acylation. Part XV. The spontaneous and acid-catalysed hydrolysis of diacetyl sulphide. J. Chem. Soc. (Resumed), 1057–1062, doi: 10.1039/JR9650001057 (1965).

[b36] Archer D. G. & Wang P. The Dielectric Constant of Water and Debye‐Hückel Limiting Law Slopes. J. Phys. Chem. Ref. Data 19, 371–411 (1990).

[b37] Jencks W. P., Cordes S. & Carriuolo J. The free energy of thiol ester hydrolysis. J. Biol. Chem. 235, 3608–3614 (1960). 13789811

[b38] Guynn R. W., Webster L. T. & Veech R. L. Equilibrium constants of the reactions of acetyl coenzyme A synthetase and the hydrolysis of adenosine triphosphate to adenosine monophosphate and inorganic pyrophosphate. J. Biol. Chem. 249, 3248–3254 (1974). 4275341

[b39] Lang S. Q., Butterfield D. A., Schulte M., Kelley D. S. & Lilley M. D. Elevated concentrations of formate, acetate and dissolved organic carbon found at the Lost City hydrothermal field. Geochim. Cosmochim. Acta 74, 941–952, doi: 10.1016/j.gca.2009.10.045 (2010).

[b40] Holm N. G. & Charlou J. L. Initial indications of abiotic formation of hydrocarbons in the Rainbow ultramafic hydrothermal system, Mid-Atlantic Ridge. Earth Planet. Sci. Lett. 191, 1–8 (2001).

[b41] McCollom T. M. & Seewald J. S. Experimental constraints on the hydrothermal reactivity of organic acids and acid anions: I. Formic acid and formate. Geochim. Cosmochim. Acta 67, 3625–3644 (2003).

[b42] Fu Q., Sherwood Lollar B., Horita J., Lacrampe-Couloume G. & Seyfried W. E. Jr Abiotic formation of hydrocarbons under hydrothermal conditions: Constraints from chemical and isotope data. Geochim. Cosmochim. Acta 71, 1982–1998, doi: 10.1016/j.gca.2007.01.022 (2007).

[b43] Proskurowski G. *et al.* Abiogenic hydrocarbon production at Lost City hydrothermal field. Science 319, 604–607, doi: 10.1126/science.1151194 (2008). 18239121

[b44] Takano Y. *et al.* Vertical distribution of amino acids and chiral ratios in deep sea hydrothermal sub-vents of the Suiyo Seamount, Izu-Bonin Arc, Pacific Ocean. Org. Geochem. 35, 1105–1120, doi: 10.1016/j.orggeochem.2004.06.007 (2004).

[b45] Reeves E. P., McDermott J. M. & Seewald J. S. The origin of methanethiol in midocean ridge hydrothermal fluids. Proc. Nat. Acad. Sci. USA 111, 5474–5479 (2014). 24706901 10.1073/pnas.1400643111 PMC3992694

[b46] Von Damm K. L. Seafloor hydrothermal activity: black smoker chemistry and chimneys. Annu. Rev. Earth Planet. Sci. 18, 173–204 (1990).

[b47] Haynes W. M. & Lide D. R. Handbook of chemistry and physics. Vol. 92 (CRC Press, 2011).

[b48] Walker J. C. G. & Brimblecombe P. Iron and sulfur in the pre-biologic ocean. Precambrian Res. 28, 205–222, doi: 10.1016/0301-9268(85)90031-2 (1985). 11539662

[b49] McCollom T. M. In Carbon in Earth Vol. 75 Reviews in Mineralogy & Geochemistry (eds Hazen R. M., Jones A. P. & Baross J. A.) 467–494 (2013).

[b50] Flamholz A., Noor E., Bar-Even A. & Milo R. eQuilibrator—the biochemical thermodynamics calculator. Nuc. Acids Res. 40, D770–D775 (2012). 10.1093/nar/gkr874 PMC3245061 22064852

[b51] Wiesli R. A., Beard B. L. & Johnson C. M. Experimental determination of Fe isotope fractionation between aqueous Fe(II), siderite and “green rust” in abiotic systems. Chem. Geol. 211, 343–362, doi: 10.1016/j.chemgeo.2004.07.002 (2004).

[b52] Trail D., Watson E. B. & Tailby N. D. The oxidation state of Hadean magmas and implications for early Earth’s atmosphere. Nature 480, 79–82 (2011). 22129728 10.1038/nature10655

[b53] Baross J. & Hoffman S. Submarine hydrothermal vents and associated gradient environments as sites for the origin and evolution of life. Origins Life Evol. B. 15, 327–345, doi: 10.1007/BF01808177 (1985).

[b54] Lazcano A. & Miller S. L. How long did it take for life to begin and evolve to cyanobacteria? J. Mol. Evol. 39, 546–554 (1994). 11536653 10.1007/BF00160399

[b55] Kelemen P. B. & Manning C. E. Reevaluating carbon fluxes in subduction zones, what goes down, mostly comes up. Proc. Nat. Acad. Sci. USA 112, E3997–E4006 (2015). 26048906 10.1073/pnas.1507889112 PMC4522802

[b56] Canfield D. E. The evolution of the Earth surface sulfur reservoir. Am. J. Sci. 304, 839–861 (2004).

[b57] Holland H. D. The chemical evolution of the atmosphere and oceans (Princeton University Press, 1984).

[b58] Schulte M. D. & Rogers K. L. Thiols in hydrothermal solution: standard partial molal properties and their role in the organic geochemistry of hydrothermal environments. Geochim. Cosmochim. Acta 68, 1087–1097, doi: 10.1016/j.gca.2003.06.001 (2004).

[b59] Martens C. S. Generation of short chain acid anions in hydrothermally altered sediments of the Guaymas Basin, Gulf of California. Appl. Geochem. 5, 71–76, doi: 10.1016/0883-2927(90)90037-6 (1990).

[b60] Belsky A. J., Maiella P. G. & Brill T. B. Spectroscopy of hydrothermal reactions 13. Kinetics and mechanisms of decarboxylation of acetic acid derivatives at 100−260 °C under 275 bar. J. Phys. Chem. A 103, 4253–4260, doi: 10.1021/jp984122d (1999).

[b61] Heinen W. & Lauwers A. M. Organic sulfur compounds resulting from the interaction of iron sulfide, hydrogen sulfide and carbon dioxide in an anaerobic aqueous environment. Origins Life Evol. Biospheres 26, 131–150, doi: 10.1007/bf01809852 (1996). 11536750

[b62] Baaske P. *et al.* Extreme accumulation of nucleotides in simulated hydrothermal pore systems. Proc. Nat. Acad. Sci. USA 104, 9346–9351, doi: 10.1073/pnas.0609592104 (2007). 17494767 PMC1890497

[b63] Nitschke W. & Russell M. J. Beating the acetyl coenzyme A-pathway to the origin of life. Phil. Trans. Roy. Soc. London B: Biol. Sci. 368, doi: 10.1098/rstb.2012.0258 (2013). PMC3685460 23754811

[b64] Hill J. Sulfur and the Origins of Life. Master of Science thesis, University of Canterbury, (2000).

[b65] Ulijn R. V., Moore B. D., Janssen A. E. & Halling P. J. A single aqueous reference equilibrium constant for amide synthesis–hydrolysis. J. Chem. Soc. Perk. Trans. 2, 1024–1028 (2002).

[b66] Morse J. W. & Mackenzie F. T. Hadean ocean carbonate geochemistry. Aquat. Geochem. 4, 301–319, doi: 10.1023/a:1009632230875 (1998).

[b67] Sleep N. H. The Hadean-Archaean environment. Cold Spring Harbor Perspectives in Biology 2, a002527 (2010). 20516134 10.1101/cshperspect.a002527 PMC2869525

[b68] Sillén L. G. The ocean as a chemical system. Science 156, 1189–1197, doi: 10.1126/science.156.3779.1189 (1967). 17792775

[b69] Glein C. R., Baross J. A. & Waite J. H. The pH of Enceladus’ ocean. Geochim. Cosmochim. Acta 162, 202–219 (2015).

[b70] Hussmann H., Sohl F. & Spohn T. Subsurface oceans and deep interiors of medium-sized outer planet satellites and large trans-neptunian objects. Icarus 185, 258–273, doi: 10.1016/j.icarus.2006.06.005 (2006).

[b71] Mustard J. F. *et al.* Hydrated silicate minerals on Mars observed by the Mars Reconnaissance Orbiter CRISM instrument. Nature 454, 305–309 (2008). 18633411 10.1038/nature07097

[b72] Toupance G., Raulin F. & Buvet R. Formation of prebiochemical compounds in models of the primitive Earth’s atmosphere. Origins Life 6, 83–90 (1975). 10.1007/BF01372392 168541

[b73] Rosenthal D. & Taylor T. I. A study of the mechanism and kinetics of the thioacetamide hydrolysis reaction. J. Am. Chem. Soc. 79, 2684–2690, doi: 10.1021/ja01568a007 (1957).

[b74] Nagai Y., Morooka S., Matubayasi N. & Nakahara M. Mechanisms and kinetics of acetaldehyde reaction in supercritical water: Noncatalytic disproportionation, condensation, and decarbonylation. J. Phys. Chem. A 108, 11635–11643, doi: 10.1021/jp046117h (2004).

[b75] Schulte M. D. & Shock E. L. Aldehydes in hydrothermal solution: Standard partial molal thermodynamic properties and relative stabilities at high temperatures and pressures. Geochim. Cosmochim. Acta 57, 3835–3846, doi: 10.1016/0016-7037(93)90337-V (1993). 11539453

[b76] Lipmann F. Attempts to map a process evolution of peptide biosynthesis. Science 173, 875–884, doi: 10.1126/science.173.4000.875 (1971). 4937229

[b77] Cody G. *et al.* Assaying the catalytic potential of transition metal sulfides for abiotic carbon fixation Geochim. Cosmochim. Acta 68, 2185–2196 (2003).

[b78] Holm N. G. & Andersson E. In The Molecular Origins of Life: Assembling the Pieces of the Puzzle (ed Brack Andre) (Cambridge University Press, 1998).

[b79] White R. H. Hydrolytic stability of biomolecules at high temperatures and its implication for life at 250 °C. Nature 310, 430–432 (1984). 6462230 10.1038/310430a0

[b80] Koshland D. E. Effect of catalysts on the hydrolysis of acetyl phosphate. Nucleophilic displacement mechanisms in enzymatic reactions 1. J. Am. Chem. Soc. 74, 2286–2292, doi: 10.1021/ja01129a035 (1952).

[b81] Mattos M. & Bernini R. B. The reaction of (R)-limonene with S-thioacids. J. Braz. Chem. Soc. 18, 1068–1072 (2007).

[b82] Russell M. J. *et al.* The drive to life on wet and icy worlds. Astrobiology 14, 308–343 (2014). 24697642 10.1089/ast.2013.1110 PMC3995032

[b83] Cleaves H. J. 2nd & Chalmers J. H. Extremophiles may be irrelevant to the origin of life. Astrobiology 4, 1–9, doi: 10.1089/153110704773600195 (2004). 15104899

[b84] Douville E. *et al.* The rainbow vent fluids (36°14′N, MAR): the influence of ultramafic rocks and phase separation on trace metal content in Mid-Atlantic Ridge hydrothermal fluids. Chem. Geol. 184, 37–48, doi: 10.1016/S0009-2541(01)00351-5 (2002).

[b85] Von Damm K. L. *et al.* Chemistry of submarine hydrothermal solutions at 21°N, East Pacific Rise. Geochim. Cosmochim. Acta 49, 2197–2220, doi: 10.1016/0016-7037(85)90222-4 (1985).

[b86] Welhan J. A. & Craig H. In Hydrothermal Processes at Seafloor Spreading Centers Vol. 12 NATO Conference Series (eds Rona P. A., Boström K., Laubier L. & Smith K. L. Jr.) Ch. 17, 391–409 (Springer US, 1983).

[b87] Kelley D. S. *et al.* An off-axis hydrothermal vent field near the Mid-Atlantic Ridge at 30°N. Nature 412, 145–149 (2001). 11449263 10.1038/35084000

[b88] Tivey M. K. Generation of seafloor hydrothermal vent fluids and associated mineral deposits. Oceanogr. 20, 50–65, doi: 10.5670/oceanog.2007.80. (2007).

